# Sequence similarity is more relevant than species specificity in probabilistic backtranslation

**DOI:** 10.1186/1471-2105-8-58

**Published:** 2007-02-21

**Authors:** Alfredo Ferro, Rosalba Giugno, Giuseppe Pigola, Alfredo Pulvirenti, Cinzia Di Pietro, Michele Purrello, Marco Ragusa

**Affiliations:** 1 Dipartimento di Matematica e Informatica, Università di Catania, Viale A. Doria 6, I-95125 Catania, Italy; 2 Dipartimento di Scienze Biomediche, Università di Catania, Via S. Sofia 87, I-95125 Catania, Italy

## Abstract

**Background:**

Backtranslation is the process of decoding a sequence of amino acids into the corresponding codons. All synthetic gene design systems include a backtranslation module. The degeneracy of the genetic code makes backtranslation potentially ambiguous since most amino acids are encoded by multiple codons. The common approach to overcome this difficulty is based on imitation of codon usage within the target species.

**Results:**

This paper describes EasyBack, a new parameter-free, fully-automated software for backtranslation using Hidden Markov Models. EasyBack is not based on imitation of codon usage within the target species, but instead uses a sequence-similarity criterion. The model is trained with a set of proteins with known cDNA coding sequences, constructed from the input protein by querying the NCBI databases with BLAST. Unlike existing software, the proposed method allows the quality of prediction to be estimated. When tested on a group of proteins that show different degrees of sequence conservation, EasyBack outperforms other published methods in terms of precision.

**Conclusion:**

The prediction quality of a protein backtranslation methis markedly increased by replacing the criterion of most used codon in the same species with a Hidden Markov Model trained with a set of most similar sequences from all species. Moreover, the proposed method allows the quality of prediction to be estimated probabilistically.

## Background

In natural systems, proteins are synthesized using template mRNA derived from molecules transcribed from the encoding genes. Backtranslation (reverse translation) reverses the normal flow of information, exploiting the primary structure of a protein to deduce the nucleotide sequence of the encoding mRNA. Backtranslation tools are basic to the construction of synthetic DNA segments (gene design systems) [1]. Such systems use suitable modules to optimize backtranslated segments to be used for expression by a host organism, or to be changed completely to accommodate various constraints [2-4].

The degeneracy of the genetic code makes backtranslation potentially ambiguous since most amino acids are encoded by multiple codons. Extensive studies have been conducted on synonymous codon usage in different species and its influence in biological processes such as structure prediction [5-9].

The approach to backtranslation common to all commercial and non-commercial software (BBOCUS [10], BACKTRANSEQ of the EMBOSS software suite [11]) is based on imitation of codon usage within the target species. For some of these methods, expert supervision is required to construct the codon usage tables. Several methods are based on the hypothesis that specific genomic contexts may influence codon usage (TIP [12,13], LBT [14]). The genetic algorithm TIP uses a set of "coding statistics", whereas LBT exploits Multiple Sequence Alignment (MSA) of the class of proteins under analysis. Both software packages give high-precision results. However, their users must set a number of parameters if the results are to be reliable.

In this paper, a parameter-free and fully-automated software called EasyBack is proposed. Given an amino acid sequence as input, EasyBack tries to reconstruct the codon usage of the gene under analysis using a Hidden Markov Model (HMM) [15]. The model is trained with an "input-driven" training set. This set of proteins is constructed from the input protein by querying the NCBI [16] databases with BLAST. The training set will be the "smallest" subset of the query output needed for HMM to make a prediction. The prediction is made by classical Viterbi or posterior decoding algorithms [15]. Prediction quality can be estimated by analyzing the posterior and forward probabilities. Experiments on eukaryotic and prokaryotic proteins showing different degrees of conservation demonstrate that EasyBack outperforms TIP and BACKTRANSEQ in terms of precision (i.e. number of codons properly decoded). Consequently, sequence similarity applied to all species yields better results than imitation of codon usage within the target species.

## Implementation

**Figure 1:**
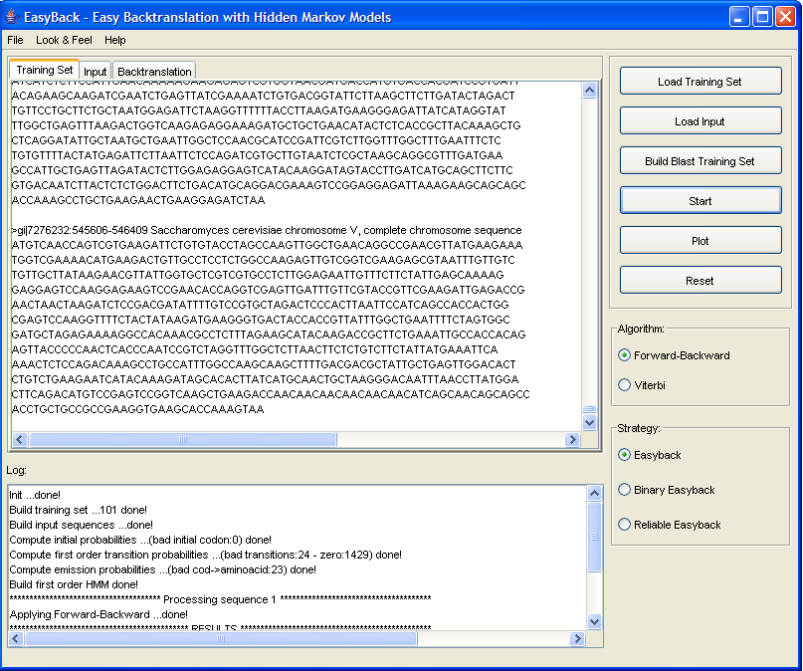
EasyBack main application interface.

**Figure 2:**
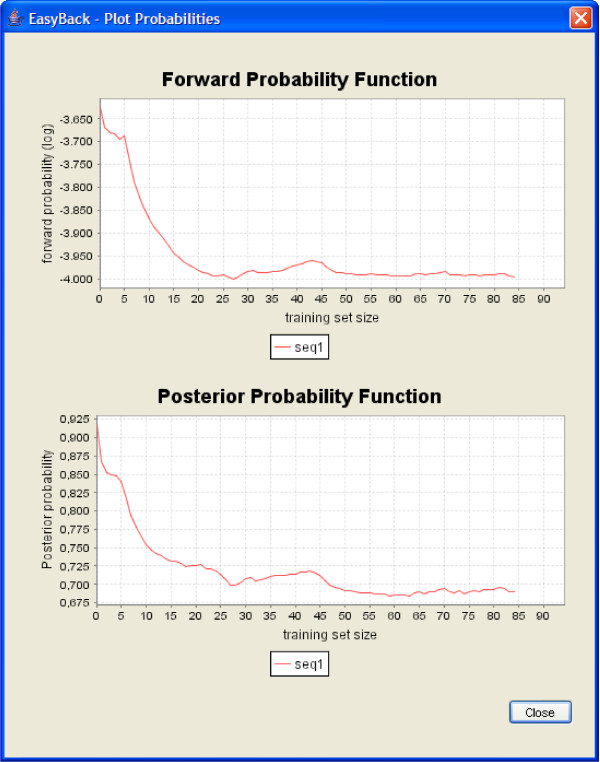
**EasyBack interface (probabilities graphs)**. EasyBack computes a forward and posterior probabilities plots. Forward probability function can suggest the smallest size of the training set needed for a reliable prediction. Oscillation of the posterior probability indicates that a low percentage of amino acids has been correctly decoded.

EasyBack is an Open-Source backtranlsation tool implemented as a Java application. The Java package JFreeChart [17] has been used to depict graphs (see Figure 1 and Figure 2 for EasyBack application interface). EasyBack system is based on a Hidden Markov Model (briefly described below).

### Hidden Markov Models overview

A Hidden Markov Model (HMM) is composed of:

1. A set *S *= {*S*_1_, *S*_2_,…,*S*_*N*_} of hidden states. The state at time *t *is denoted by *q*_t_;

2. A set *V *= {*V*_1_, *V*_2_,…,*V*_*M*_} of observation symbols;

3. A state transition probability distribution *A*, represented as an *N *× *N *matrix where the generic element is *a*_*ij *_= *P*[*q*_*t*+1 _= *S*_*j*_|*q*_*t *_= *S*_*i*_], the probability that *S*_*j *_is the state at time *t *+ 1 if *S*_*i *_is the state at a previous time *t*. Notice that *a*_*ij *_≥ 0 and ∑j=1Naij=1
 MathType@MTEF@5@5@+=feaafiart1ev1aaatCvAUfKttLearuWrP9MDH5MBPbIqV92AaeXatLxBI9gBaebbnrfifHhDYfgasaacH8akY=wiFfYdH8Gipec8Eeeu0xXdbba9frFj0=OqFfea0dXdd9vqai=hGuQ8kuc9pgc9s8qqaq=dirpe0xb9q8qiLsFr0=vr0=vr0dc8meaabaqaciaacaGaaeqabaqabeGadaaakeaadaaeWaqaaiabdggaHnaaBaaaleaacqWGPbqAcqWGQbGAaeqaaOGaeyypa0JaeGymaedaleaacqWGQbGAcqGH9aqpcqaIXaqmaeaacqWGobGta0GaeyyeIuoaaaa@3955@;

4. An observation symbol probability distribution *B*, represented as an *N *× *M *matrix where the generic element is *b*_*j*_(*k*) = *P*[*V*_*k *_at *t*|*q*_*t *_= *S*_*j*_], the probability that *V*_*k *_is observed at time *t *in the hidden state *S*_*j*_;

5. An initial state distribution *π *represented as a vector of which the generic element is *π*_*i *_= *P*[*q*_1 _= *S*_*i*_], probability that the initial state is *S*_*i*_.

Given a HMM, *λ *= (*A*, *B*, *π*), three basic problems arise in real applications (see [15,18] for details).

1. Given an observation sequence *O *= *O*_1 _*O*_2_…*O*_*T *_(where each *O*_*t *_is a symbol in *V*), compute the most likely corresponding hidden state sequence *Q *= *q*_1_*q*_2_…*q*_*T*_. In this paper we deal with this problem. It can be solved by a classical Viterbi algorithm or a posterior decoding technique based on a forward-backword algorithm. Both methods are used to make prediction.

2. Given an observation sequence *O *= *O*_1 _*O*_2_…*O*_*T*_, compute the probability *P*(*O*|*λ*) of the observation *O *in the model *λ*. Together with the posterior probability, this will be used to determine the reliability of back translation.

3. Given an observation sequence *O *= *O*_1 _*O*_2_…*O*_*T*_, tune the model's parameters in order to maximize *P*(*O*|*λ*).

### EasyBack

Let *q *be an input sequence with unknown backtranslation, and let *T *be the training set of sequences. The set of states of the HMM will be *S *= {*s*_1_, *s*_2_,…,*s*_64_} of all possible codons. A transition from state *s*_*i *_to state *s*_*j *_corresponds to a pair of consecutive amino acids coded by *s*_*i *_and *s*_*j*_, respectively. The alphabet of the HMM comprises the 20 amino acids. The transition probability of two codons *s*_*i *_and *s*_*j *_is the number of occurrences of the pair of consecutive codons "*s*_*i*_*s*_*j*_" in the training set divided by the number of occurrences of *s*_*i *_not followed by a stop codon. The probability that a codon *s*_*i *_generates an amino acid *a *(emission probability) is the number of times *a *is decoded by *s*_*i *_in the training set divided by the number of occurrences of *a *in such a set. Since stop codons do not encode an amino acid, then their emission probability is zero.

Three different ways to apply EasyBack have been considered: *simple *(using the simple BLAST-similarity strategy), *binary *(trying to reduce the training set size), *reliable *(using forward and posterior probability diagrams to optimize prediction quality).

**Figure 3:**
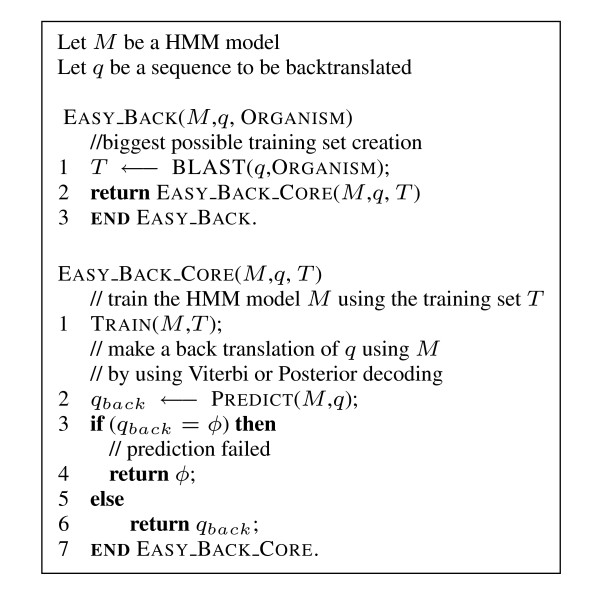
**Core EasyBack algorithm**. Description of EasyBack algorithm.

EasyBack uses a protein sequence to deduce cDNA (nucleotide) sequences from NCBI database. In the *simple *strategy, given a query *q*, a BLAST query to NCBI is performed with input *q*. Let *T *be the output of the query. The model is trained with *T *and eventually a prediction is returned (see Figure 3 for the pseudo-code and Figure 1 for the application interface).

In the *binary *strategy, the model is trained with the smallest set needed to make a prediction. More precisely, a BLAST query is submitted to NCBI with input *q *and the best 100 distinct matches are selected. Let *T*_1 _be such a set of sequences. If the HMM fails to make a prediction with training set *T*_1 _then add to *T*_1 _the next best 100 (the choice of 100 matches was sufficient to make a prediction in all experimental groups of proteins, chosen with variable degree of conservation) matches, and so on until a prediction can be made. The failure condition is that for a given amino acid in the input sequence, the corresponding entry in the transition probability matrix is undefined.

**Figure 4:**
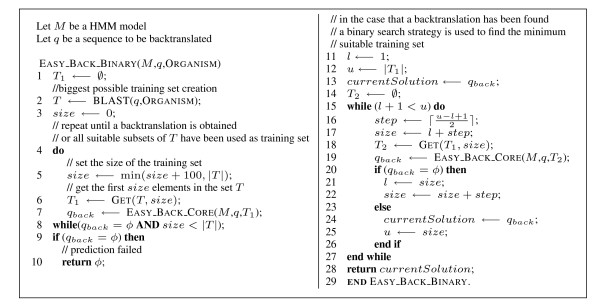
**Binary EasyBack algorithm**. Description of EasyBack algorithm with the smallest training set needed for the model to make a prediction.

Otherwise, if the HMM is able to make a prediction with *T*_1_, then repeat the process using the best |*T*_1_|/2 matches. Let *T*_2 _be such a set. If the HMM fails with *T*_2 _then amend *T*_2 _to be the best (|*T*_1_| + |*T*_2_|)/2 matches. If *T*_2 _succeeds then amend it to the best |*T*_2_|/2 matches. This binary search process stops in *O*(log(|*T*_1_|) producing the final HMM prediction, which is the approximate backtranslation of the input *q *(see Figure 4 for the pseudo-code and Figure 1 for the application interface).

**Figure 5:**
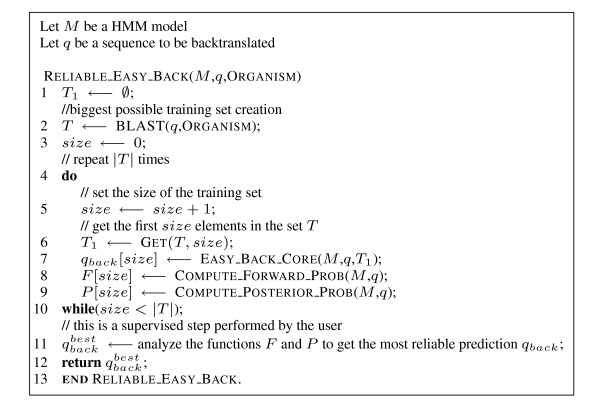
**Reliable EasyBack algorithm**. Description of EasyBack algorithm in which forward and posterior probabilities are stored and analyzed to determine the most reliable backtranslation.

In the *reliable *strategy, a probabilistic estimation of prediction quality is made. Given a query *q*, a BLAST query to NCBI is performed with input *q*. Let *T *be the output of the query. The model is trained |*T*| times, starting with a training set that contains only the first element of *T *and adding the next element of *T *iteratively. A prediction is made for each iteration and the forward and posterior probabilities are computed. The graphs of these probabilities are analyzed and the most reliable prediction is selected (see Figure 5 for the pseudo-code and Figure 1 and Figure 2 for the application interface). More precisely, the forward probability function can suggest the smallest size of the training set needed for a reliable prediction. Finally, unusual oscillation of the posterior probability indicates that a low percentage of amino acids has been correctly decoded.

## Results and Discussion

### Approach

EasyBack is a backtranslation tool based on a Hidden Markov Model trained with an "input-driven" training set. A HMM (for more details see [15]) describes a system comprising *N *different hidden states with transition probabilities associated with each pair of states. The states generate observable symbols with probabilities computed from a training set. Given a series of observable symbols, the HMM can decode the most probable corresponding sequence of hidden states. In the proposed model, the hidden states are all possible codons and the observable symbols are the amino acids decoded by them. The transition probability of two codons *s*_*i *_and *s*_*j *_is the number of occurrences of the pair of consecutive codons *s*_*i*_*s*_*j *_in the training set divided by the number of the occurrences of *s*_*i *_not followed by a stop codon. The probability that a codon *s*_*i *_generates an amino acid *a*, the emission probability, is the number of times *a *is decoded by *s*_*i *_in the training set divided by the number of occurrences of *a *in such a set. The training set is constructed by applying a criterion of similarity between the input protein sequence *q *to be backtranslated and sequences in the NCBI database. More precisely, a BLAST query is submitted to NCBI with input *q *and the "smallest" subset of the query output that enables HMM to make a prediction is chosen as the training set. Therefore, the size of the training set is related to the number of non-zero values contained in the matrix of transition probabilities. More precisely, when the system fails to make a prediction, this means that at least one necessary transition probability value in the matrix is zero. In this case the training set must be enlarged with more sequences. The backtranslation of *q *is obtained by applying either the Viterbi or the Forward-Backward algorithm to the model (posterior decoding) [15]. One useful aspect of HMM is the ability to choose several strategies for posterior estimation of the reliability of a prediction (e.g. see [19] for multiple sequence alignment). The forward probability function can suggest the size of the smallest training-set needed for reliable prediction. The higher this probability, the better the prediction obtained from the training set. Furthermore, analysis of the posterior probability allows the quality of prediction to be established. More precisely, if the probability oscillates unusually as a function of the training set size, then a low percentage of amino acids has been correctly decoded.

### Test sets

To assess the efficiency of the proposed method, a set of *Homo sapiens *and prokaryotic proteins with various degrees of primary structure conservation was backtranslated (the conservation degree of the experimental set of proteins was obtained by calculating the proportion of amino acid sites at which the two sequences under study were identical [20]):

• Proteins present in all eukaryotes: histone H4 (HIST4H4) (97.7%), SOD2 [21] (67.1%), NP_006752.1 (YWHAG), and NP_036611.2 (YWHAE).

• Proteins present in all metazoa: TBP (81%), fibrinopeptide (FGA) (60.9%), and myosin (MYH9) (59.7%).

• Proteins present only in vertebrates: tyrosinase (TYR) (73.9%), alpha globin (HBA2) (65.2%), and beta globin (HBB) (62.5%).

• Proteins present in prokaryotes: NP_438418.1 (*Haemophilus influenzae*).

• Ribosomal proteins: ribosomal protein L36a (RPL36A), ribosomal protein S6 kinase, 90 kDa (RPS6KA1).

EasyBack was trained with three different kinds of training sets:

• *BLAST All-Species*. This training set was obtained by querying the NCBI all-species database with the input sequence, using BLAST;

• *Random Species-Specific*. This training set was obtained by randomly choosing sequences that belong to species expressing the input protein;

• *BLAST Invertebrates*: for *Homo sapiens *proteins, the training set was obtained by querying the NCBI invertebrates database with the input sequence, using BLAST;

• *BLAST Eukaryote*: for prokaryotic proteins, the training set was obtained by querying the NCBI eukaryote database with the input sequence, using BLAST.

Since biological sequence databases are notorious for having multiple copies of sequence fragments in different entries, homologous found with BLAST that contained portions of the sequence under test were carefully manually eliminated to make the testing process fair. On the other hand this manual filtering is not necessary for an unknown input amino acid sequence. This was the reason for not considering *BLAST Species-Specific *training sets (insufficient numbers of sequences were returned). *BLAST Species-Specific *training set was obtained by querying with the input sequence, using BLAST, the sequences of NCBI database belonging to species of the input protein.

**Figure 6:**
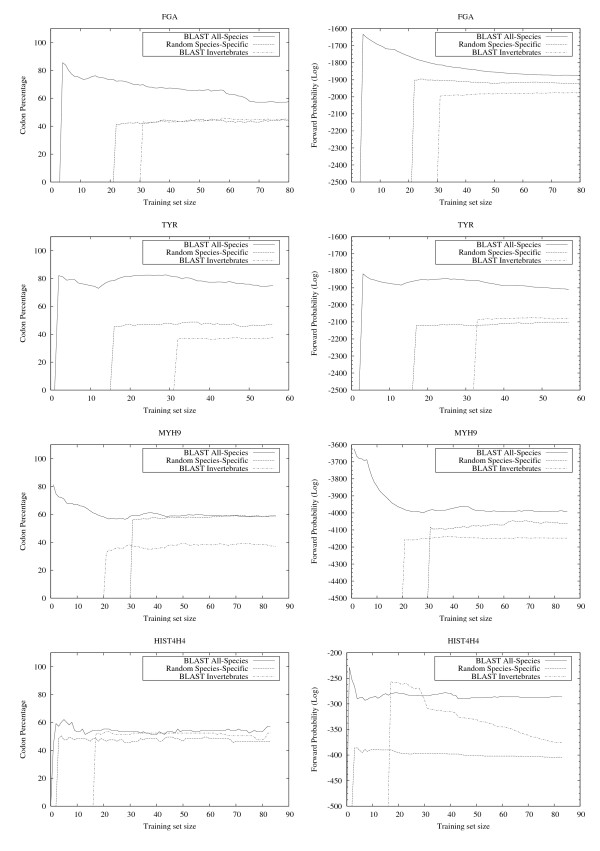
**EasyBack Performace Analysis on FGA, TYR, MYH9 and HIST4H4**. Left column: EasyBack prediction performance (percentage of amino acids correctly decoded). Input proteins are: FGA (fibrinopeptide), TYR (tyrosinase), MYH9 (myosin), HIST4H4 (histone H4). Right column: forward probability. The quality of prediction using *BLAST All-Species *training sets is higher than both *Random Species-Specific *(sequences belonging to the same organism) and *BLAST Invertebrates *(distant organisms). The forward probability can be used to estimate the best training set size. In almost all cases a high forward probability corresponds to a high quality backtranslation.

**Figure 7:**
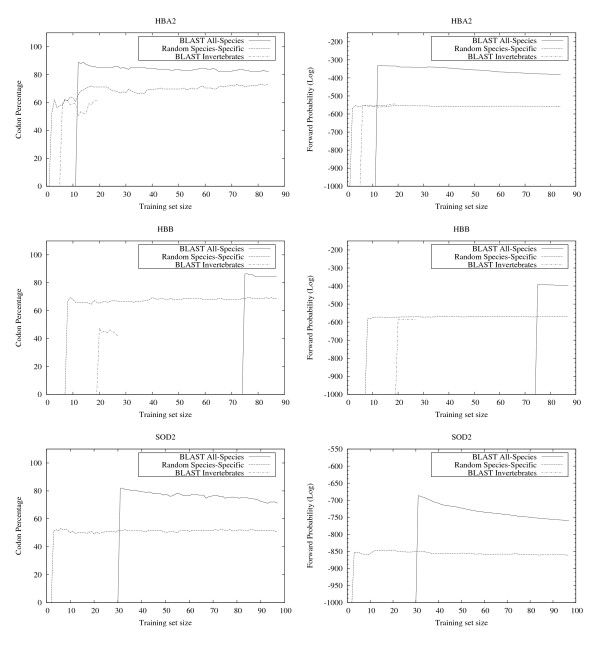
**EasyBack Performace Analysis on HBA2, HBB and SOD2**. Left column: EasyBack prediction performance (percentage of amino acids correctly decoded). Right column: forward probability. (See caption of Figure 6). Input proteins are: HBA2 (alpha globin), HBB (beta globin), and SOD2.

**Figure 8:**
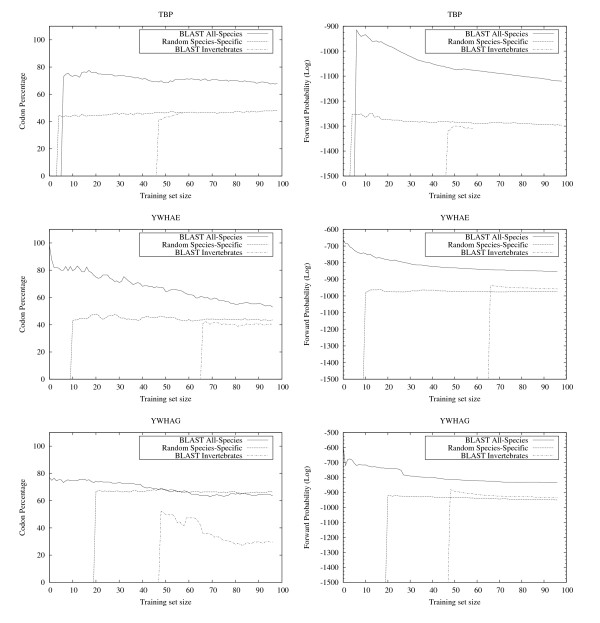
**EasyBack Performace Analysis on YWHAE, YWHAG and TBP**. Left column: EasyBack prediction performance (percentage of amino acids correctly decoded). Right column: forward probability. (See caption of Figure 6). Input proteins are: YWHAE (NP_036611.2), YWHAG (NP_006752.1), and TBP.

**Figure 9:**
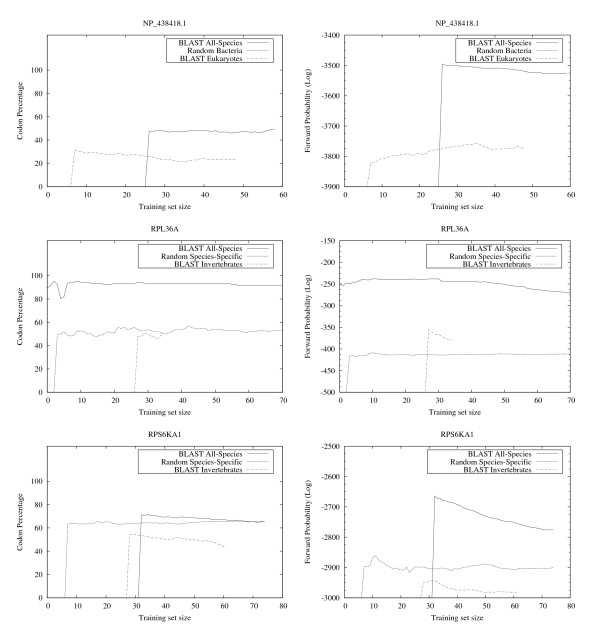
**EasyBack Performace Analysis on NP_438418.1, RPL36A and RPS6KA1**. Left column: EasyBack prediction performance (percentage of amino acids correctly decoded). Right column: forward probability. The quality of prediction using *BLAST All-Species *training sets is higher than both *Random Species-Specific *(sequences belonging to the same organism) and *BLAST Invertebrates *for RPL36A and RPS6KA1 and *BLAST Eukaryotes *for NP_438418.1 (distant organisms). The forward probability can be used to estimate the best training set size. In almost all cases a high forward probability corresponds to a high quality backtranslation. Input proteins are: NP_438418.1 (from *Haemophilus influenzae *species), RPL36A (ribosomal protein L36a), RPS6KA1 (ribosomal protein S6 kinase, 90 kDa).

The results show that EasyBack clearly performs better, in terms of percentage of correctly decoded codons, when trained with *BLAST All-Species *(see left column of Figures 6, 7, 8, 9). However, the prediction quality is degraded if sequences belonging to a distantly-related organism are chosen as training set (e.g. Homo sapiens SOD2 on Invertebrates data set, Bacteria NP_438418.1 on Eukaryotes). Moreover, HMM trained only with sequences from organisms other than the one from which the sequence under test was obtained showed no decrease in prediction quality (these experiments are not reported here since the performance was very close to that with *BLAST All-Species*).

**Figure 10:**
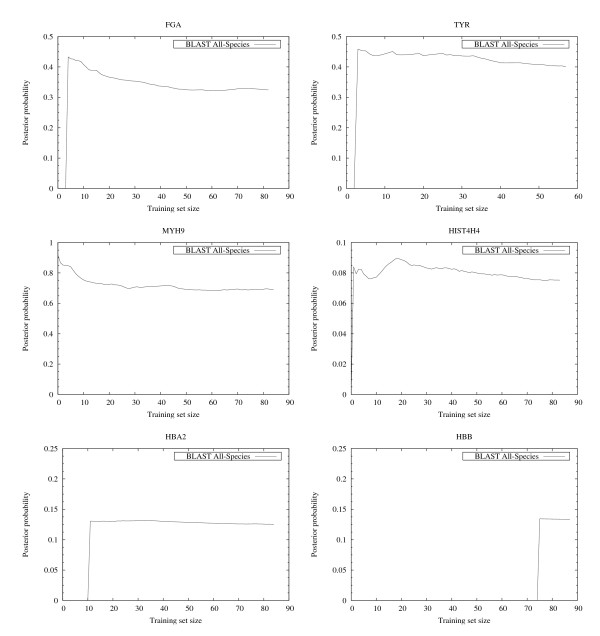
**EasyBack posterior probabilities**. The oscillating behavior of the posterior probability of histone H4 corresponds empirically to the low quality of its backtranslation (see the graph reporting the correctly decoded codon percentage of HIST4H4 in Figure 6).

**Figure 11:**
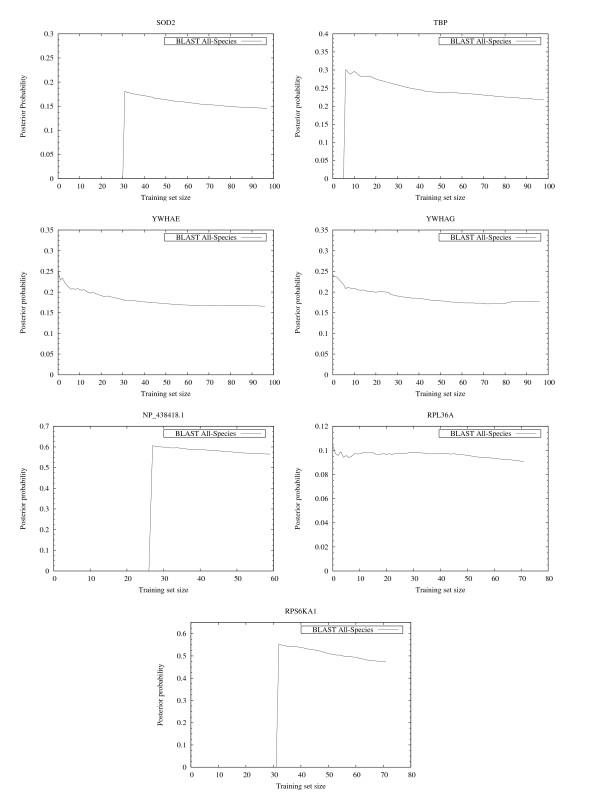
**EasyBack posterior probabilities**. In these cases non-oscillating behavior is reported. This is associated with high quality backtranslation (see graphs reporting their correctly decoded codon percentages in Figure 7, Figure 8 and Figure 9).

**Figure 12:**
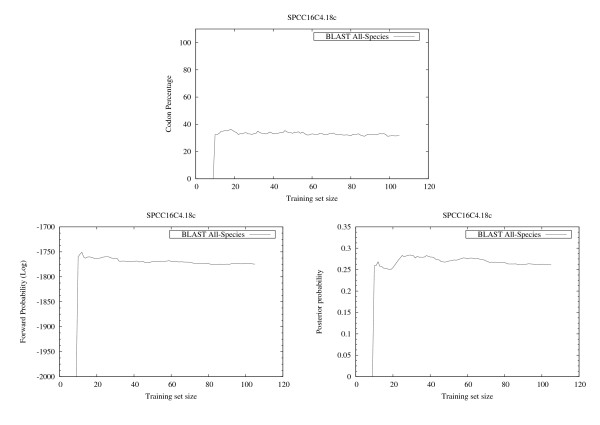
**EasyBack Performace Analysis on SPCC16C4.18c**. SPCC16C4.18c shows oscillating posterior probabilities corresponding to low quality decoding.

The results summarized in the right column of Figure 6 and in Figure 7, 8, 9 show that, for all cases except HISTH4, the most reliable prediction is obtained using the training set with the highest forward probability. Moreover, the quality of the prediction can be estimated by analyzing posterior probability. The unusual oscillation of posterior probability in Figure 10 and 11 for Histon H4 and Figure 12 for SPCC16C4.18c from *Schizosaccharomyces pombe *indicates that only a low percentage of the amino acids were correctly decoded.

Despite experiments show that similarity is more relevant than species specificity, a reliable prediction depends on how the training set is "biologically related" to the input sequence. Acquiring knowledge able to correlate the quality of prediction to the composition of the training set is a hard problem and will be subject of future research. For example, prediction quality for RPL36A was significantly higher than Hist4H4. On the other hand for both proteins prediction quality did not decrease by augmenting the training set. The mathematical explanation of this phenomenon can by expressed in terms of a better agrement in RPL36A vs Hist4H4 in the Markovian codon transition/emission probabilities among the elements in the training set. In any case EasyBack is able to estimate prediction quality and optimal training set size by forward and posterior probability computation respectively.

### Comparisons

**Figure 13:**
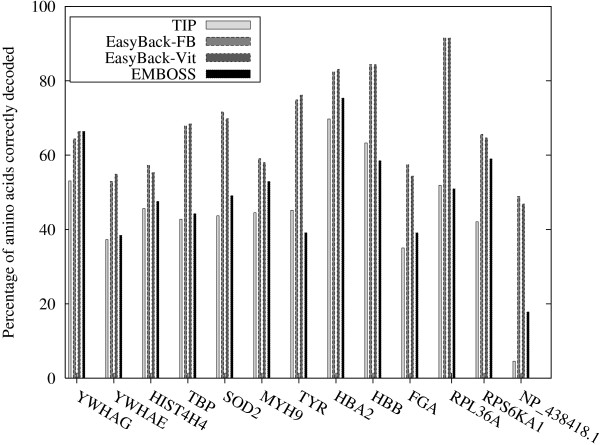
**EasyBack vs TIP and BACKTRANSEQ**. Performance of EasyBack compared with TIP and BACK-TRANSEQ based on percentages of amino acids correctly decoded. Easyback and TIP were tested using *BLAST All-Species *training sets. BACKTRANSEQ used Species-Specific training sets. For all systems each training set comprised 100 sequences. Classical Viterbi algorithm (EasyBack-Vit) and a posterior decoding technique based on a forward-backword algorithm (EasyBack-FB) were used to make predition.

**Figure 14:**
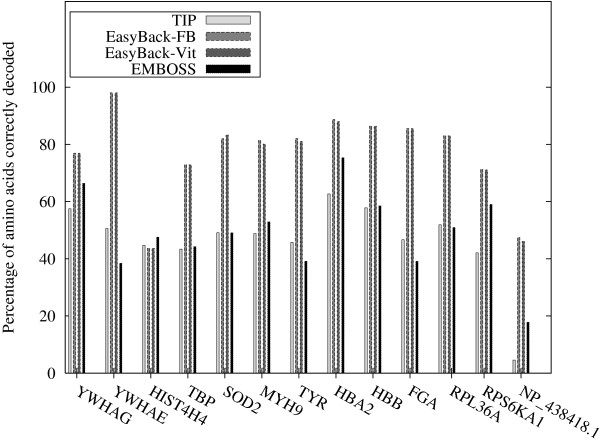
**EasyBack vs TIP and BACKTRANSEQ (binary search strategy)**. Performance of EasyBack compared with TIP and BACKTRANSEQ based on the percentages of amino acids correctly decoded. The training set is the minimal subset of the query output sufficient to make a prediction obtained by a binary search strategy. EasyBack-FB and EasyBack-Vit denote Forward-Backward and Viterbi, respectively.

EasyBack was successfully compared with TIP [12] and BACKTRANSEQ [11] (see Table 1 for details). In all the experiments described below, the same training sets obtained using *BLAST All-Species *criteria for EasyBack and TIP were used. In contrast BACKTRANSEQ was designed to be used only with *Species-Specific *training sets (each amino acid is decoded by the most frequent coding codon in the species). In the first comparison (Figure 13, Table 2) a training set of a fixed size (100 sequences) was used. In the second comparison (Figure 14, Table 3), the binary strategy procedure described in the Methods section was applied to generate the "smallest" training set needed for prediction. EasyBack, TIP and BACKTRANSEQ were also compared using species-specific training sets. For TIP and EasyBack, the training sets were chosen randomly; for BACKTRANSEQ, the most frequent codon criterion was used. The results show that species-specific training sets give lower-quality predictions. Once again, EasyBack outperformed TIP and BACKTRANSEQ. Moreover, a statistical analysis was performed to support the quality of EasyBack predictions. Table 4 contains Friedman Rank test for all pairwise comparisons of EasyBack, TIP and BACKTRANSEQ. Moreover, a statistical analysis was performed to support the quality of EasyBack predictions. Table 4 contains Friedman rank test for all pairwise comparisons of EasyBack, TIP and BACKTRANSEQ.

**Table 1:** Comparison of existing backtranslation tools.

	Training Set	Learning Method	Unsupervised
BBOCUS [10]	Species specific	Clustering and codon frequencies	No
**EasyBack**	**Any**	**HMM**	**Yes**
BACKTRANSEQ [11]	Species specific	Codon frequencies	Yes
LBT [14]	Any	Alignment and local codon frequencies	Yes
TIP [12]	Any	Genetic algorithm and statistics	Yes

**Table 2:** Comparisons of EasyBack with TIP and BACKTRANSEQ based on percentages of amino acids correctly decoded.

**Test ID**	**EasyBack-FB**	**EasyBack-Vit**	**TIP**	**BACKTRANSEQ**
YWHAG	0.64	**0.66**	0.53	0.66
YWHAE	0.53	**0.55**	0.37	0.38
HIST4H4	**0.57**	0.55	0.46	0.48
TBP	**0.68**	**0.68**	0.43	0.44
SOD2	**0.72**	0.70	0.44	0.49
MYH9	**0.59**	0.58	0.45	0.53
TYR	0.75	**0.76**	0.45	0.39
HBA2	0.82	**0.83**	0.70	0.75
HBB	**0.84**	**0.84**	0.63	0.59
FGA	**0.57**	0.54	0.35	0.39
RPL36A	**0.92**	**0.92**	0.52	0.51
RPS6KA1	**0.66**	0.65	0.42	0.59
NP_438418.1	**0.49**	0.47	0.05	0.18

Easyback and TIP were tested using *BLAST All-Species *training sets. BACKTRANSEQ used Species-Specific training sets. For all systems, each training set comprised 100 sequences. EasyBack-FB and EasyBack-Vit denote Forward-Backward and Viterbi, respectively. See Figure 13.

**Table 3:** Comparisons of EasyBack with TIP and BACKTRANSEQ based on the percentages of amino acids correctly decoded.

**Test ID**	**EasyBack-FB**	**EasyBack-Vit**	**TIP**	**BACKTRANSEQ**
YWHAG	**0.77**	**0.77**	0.58	0.66
YWHAE	**0.98**	**0.98**	0.51	0.38
HIST4H4	0.44	0.44	0.45	**0.48**
TBP	**0.73**	**0.73**	0.43	0.44
SOD2	0.82	**0.83**	0.49	0.49
MYH9	**0.81**	0.80	0.49	0.53
TYR	**0.82**	0.81	0.46	0.39
HBA2	**0.89**	0.88	0.63	0.75
HBB	**0.86**	**0.86**	0.59	0.59
FGA	**0.86**	**0.86**	0.47	0.39
RPL36A	**0.83**	**0.83**	0.51	0.51
RPS6KA1	**0.71**	**0.71**	0.5	0.59
NP_438418.1	**0.47**	**0.46**	0.05	0.18

The training set is the minimal subset of the query output sufficient to make a prediction obtained by a binary search strategy. EasyBack-FB and EasyBack-Vit denote Forward-Backward and Viterbi, respectively. See Figure 14.

**Table 4:** Significance test for differences in experiments reported in Figures 13 and 14.

	**EasyBack-FB**	**EasyBack-Vit**	**TIP**	**BACKTRANSEQ**
**EasyBack-FB**	-	-(0.57)	+0.0003	+0.0023
**EasyBack-Vit**	-(0.29)	-	+0.0003	+0.0008
**TIP**	-0.0023	-0.0023	-	-(0.05)
**BACKTRANSEQ**	-0.0023	-0.0023	+(0.4)	-

Entries show the *p*-values indicating the significance of comparisons between two backtranslation methods using the Friedman rank test. Entries above the diagonal refer to experiments in Figure 13. Entries below the diagonal refer to experiments in Figure 14. The (+) method on the left had lower average rank (better performance); the (-) method had higher average rank (worse performance); parentheses denote non-significant *p*-values > 0.05.

## Conclusion

In this paper, a backtranslation tool using a Hidden Markov Model, trained with a set of sequences most similar to the input, has been shown to outperform other published methods. All-species similarity gives better results than species-specific similarity. Furthermore, the proposed system is parameter-free and fully automated and allows the quality of prediction to be estimated (that is a clear advantage of the proposed method).

The results demonstrate that the performance of EasyBack, in terms of the percentage of amino acids correctly decoded, is considerably better than compared systems.

## Availability and requirements

• **Project name**: EasyBack

• **Project home page**: 

• **Operating system(s)**: e.g. Platform independent

• **Programming language**: Java

• **Other requirements**: Java 1.5.0_05 or higher

• **License**: Free for academic and commercial users under the GNU Lesser General Public License (LGPL)

## Authors' contributions

CDP, MP and MR proposed the problem and provided the test input sequences. AF, RG, GP and AP designed, analyzed, implemented and tested the proposed algorithm. Each author contributed equally in writing the paper. All authors read and approved the final manuscript.  Authors of each department  are listed in alphabetic order.
